# Internal hernia through an idiopathic transverse mesocolon defect treated by laparoscopic surgery: a case report

**DOI:** 10.1016/j.ijscr.2025.111701

**Published:** 2025-07-18

**Authors:** Daisuke Inoue, Katsuji Tokuhara, Hitomi Matsuki, Ryuhei Noda, Naoki Kataoka

**Affiliations:** a Department of Surgery, Kishiwada Tokushukai Hospital, 4-27-1, Kamori-cho, Kishiwada, Osaka 596-8522, Japan; b Department of Lower Gastroenterological Surgery, Kishiwada Tokushukai Hospital, 4-27-1, Kamori-cho, Kishiwada, Osaka 596-8522, Japan

**Keywords:** Transverse mesocolon hernia, Internal hernia, Laparoscopic surgery

## Abstract

**Introduction:**

Internal hernia is a rare cause of intestinal obstruction, and transverse mesocolon hernia (TMH) is an extremely rare subtype of internal hernia.

**Presentation of case:**

We report the successful treatment of a TMH through an idiopathic transverse mesocolon defect (iTMD). A 50-year-old man was rushed to hospital with sudden onset of abdominal pain. Computed tomographic imaging showed an internal hernia with small bowel obstruction in the right upper abdomen. He underwent emergency surgery for suspected strangulated intestinal obstruction caused by internal hernia. Laparoscopic surgery revealed that the small intestine was incarcerated in the iTMD. The incarcerated small intestine was reduced without requiring resection, and the iTMD was closed with barbed suture.

**Discussion:**

TMHs are rare and often difficult to diagnose preoperatively. Early recognition using characteristic computed tomographic findings can facilitate timely laparoscopic intervention and avoid complications such as bowel necrosis.

**Conclusion:**

Idiopathic TMH should be considered as a differential diagnosis in patients with an internal hernia and no history of surgery.

## Highlights

• We report an internal hernia through an idiopathic transverse mesocolon defect.
• Our case is an extremely rare treated successfully with laparoscopic surgery.
• Prompt diagnosis and treatment are important for internal hernias.

## 1 Introduction

Internal hernias account for 0.6 %–5.8 % of all intestinal obstructions, with mesenteric hernias such as an idiopathic transverse mesocolon defect (iTMD) being a particularly rare condition that accounts for 8 % of internal hernias [1]. Transverse mesocolon hernia (TMH) through an iTMD frequently causes strangulated intestinal obstruction, which requires prompt diagnosis and treatment. However, TMH is often challenging to diagnose preoperatively, leading to delayed treatment in some cases [2]. In cases of intestinal obstruction, early diagnosis can prevent significant intestinal dilatation, allowing for a clear intra-abdominal view, which often enables completion of the procedure laparoscopically. We herein report a case of TMH through an iTMD that was successfully treated with laparoscopic surgery. This manuscript has been reported according to SCARE guidelines [3].

## 2 Case presentation

**Fig. 1:**
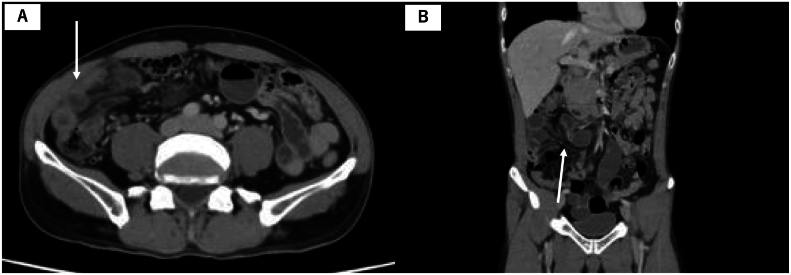
Abdominal enhanced computed tomographic images. A. There are edematous changes in the small intestine (white arrow). B. Vascular convergence and edematous changes are observed in the intestinal mesentery, and a caliber change point of the small intestine is observed nearby (white arrow).

A 50-year-old man was rushed to our hospital with sudden onset of abdominal pain. He had no history of trauma or surgery. Upon admission, the patient's vital signs were stable and his BMI was 19.8 kg/m^2^. Physical examination revealed board-like rigidity and tenderness throughout the whole abdomen. Laboratory data showed no remarkable findings. Enhanced computed tomography (CT) findings showed localized dilatation and edematous changes of the small intestine on the dorsal side of the transverse mesocolon. In addition, vascular convergence and edematous changes were observed in the mesentery of the small intestine, with a nearby caliber change point of the small intestine (Fig. 1). Therefore, he was diagnosed with strangulated intestinal obstruction caused by internal hernia.

**Fig. 2:**
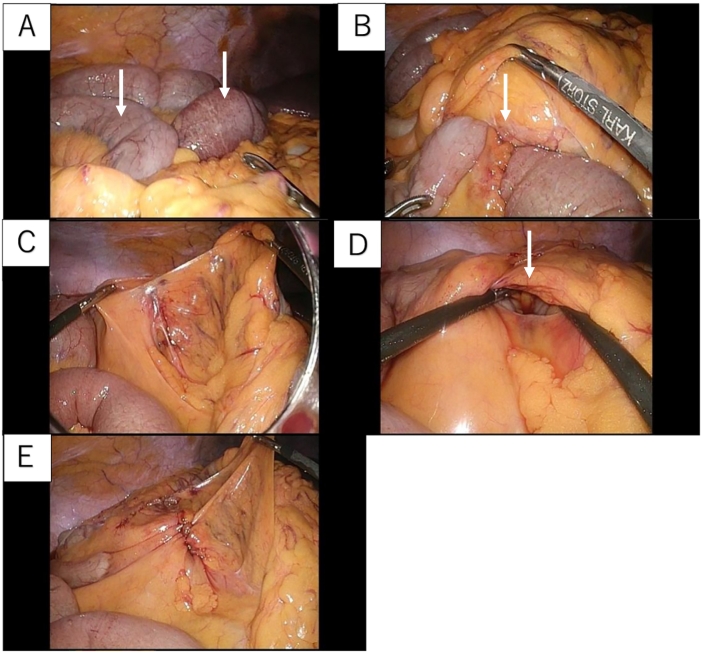
Intraoperative findings A. The small intestine has herniated cranially through the transverse colon mesentery (white arrow). B. The hernial orifice in the transverse colon mesentery with incarceration of the small intestine (white arrow). C. After reduction of the incarcerated small intestine. D. The hernial orifice in the transverse colon mesentery (white arrow). E. After continuous suture closure of the hernial orifice in the transverse colon mesentery.

Emergency laparoscopic surgery was performed using three ports. Dilated and congested small intestine was observed in the right upper abdomen (Fig. 2A). Intraoperative examination of the transverse colon cranially revealed a defect in the transverse colon mesentery, forming a hernial orifice (Fig. 2B). The small intestine had incarcerated through this orifice to the Morison's pouch. Based on these findings, we definitively diagnosed TMH through an iTMD. The incarcerated small intestine, approximately 40 cm in length, was easily reduced by gently withdrawing it from the hernial orifice (Fig. 2C) and there were no signs of necrosis. The hernial orifice (iTMD) was approximately 3 cm in diameter (Fig. 2D) and was located between the middle colic artery and the ileocolic artery; the orifice was closed using continuous sutures with 3-0 non-absorbable barbed suture (Fig. 2E). The operation time was 50 min. The patient recovered uneventfully and was discharged from hospital 5 days postoperatively.

## 3 Discussion

An internal abdominal hernia (IAH) is a protrusion of viscera through a defect or aperture, which may be either mesenteric or peritoneal. IAH is a rare cause of acute abdomen causing small bowel obstruction, accounting for only 0.6 %–5.8 % of cases [4]. The hernial orifice can be either congenital or acquired. Congenital hernial orifices include both normal apertures, such as the foramen of Winslow, and abnormal apertures resulting from anomalies in internal rotation and peritoneal attachment. Acquired hernial orifices refer to defects that are postsurgical, traumatic, or post-inflammatory in origin [2].

IAHs can be localized in several locations, with paraduodenal hernia (53 %) being the most common and transmesenteric hernia consisting of transmesocolic (8 %) and transomental (1 %–4 %) hernias being the rarest [1]. A TMH is an extremely rare type of mesenteric hernia, accounting for only 12.2 % of all mesenteric hernias [5]. TMHs are classified into two types: the bilateral type in which both the anterior and posterior lobes have defects, and the unilateral type in which only the unilateral lobe has a defect [6]. In our case, the herniated loops were not covered by a sac and the TMH was classified as the bilateral type.

The initial symptoms of IAH include abdominal distension, pain, and vomiting due to gastrointestinal obstruction. Additionally, IAHs are commonly associated with the development of strangulated gastrointestinal obstruction. Mesenteric hernias are more likely than other hernia subtypes to develop volvulus and strangulation or ischemia, with reported incidences as high as 30 % and 40 %, respectively [[7], [8], [9]]. Volvulus and strangulation or ischemia may be caused by the typically small aperture of the mesenteric hernial defect (2–5 cm) in addition to the lack of encapsulation of the herniated loops, allowing a large length of small intestine to herniate through the mesenteric defect [7]. Therefore, the time since onset has a significant impact on the incidence of intestinal ischemia.

TMH can be diagnosed based on CT imaging. However, it has been reported that only 25 % of TMHs are diagnosed preoperatively, suggesting that preoperative diagnosis is difficult. Consequently, delays before surgical intervention are not uncommon [10]. Blachar et al. reported that the typical findings of TMH on CT are i) clustering of peripheral bowel loops located outside the colon, ii) displacement of the transverse colon toward the medial, caudal, and posterior directions, and iii) displacement of the overlying omental fat [8,9]. However, further research concluded that the only statistically significant signs of TMH are relatively nonspecific findings of small intestinal dilatation with a caliber change point, clustering of small-bowel loops, and mesenteric vessel abnormalities [11].

In our case, CT imaging showed small intestinal dilation with a caliber change point in the right upper abdomen, along with convergence of the mesenteric vessels and increased fat density of the intestinal mesentery. Although the hernial orifice could not be identified, an internal hernia was diagnosed, allowing for early surgical intervention at 3 h after the onset. As early surgical intervention was carried out, we were able to reduce the incarcerated bowel and close the defect under laparoscopic surgery.

## 4 Conclusions

We report a case of idiopathic TMH treated by laparoscopic surgery. In adult patients with ileus without a history of surgery, it is necessary to consider the possibility of internal hernia, as occurred in our case. In addition, internal hernias, including TMH, require appropriate diagnosis and prompt treatment, emphasizing the importance of recognizing characteristic CT findings.

## CRediT authorship contribution statement

DI made a substantial contribution to the study conception, conducted a literature search, and drafted the manuscript. DI, KT, HM, and RN contributed to the acquisition of data. DI and KT performed the operation. DI and KT reviewed the manuscript and gave final approval for publication. All authors read and approved the final manuscript.

## Informed consent

Written informed consent was obtained from the patient for publication of this case report and accompanying images.

## Approval of the research protocol by an institutional review board and the approval number

N/A.

## Ethical approval

Ethical approval for this study was provided by the Ethical Committee of Kishiwada Tokushukai Hospital, Osaka, Japan on 15 May 2025.

## Guarantor

Katsuji Tokuhara.

## Research registration number

N/A.

## Funding

The authors declare that they have no external sources of funding.

## Declaration of competing interest

The authors declare that they have no conflict of interests.
